# Integration of AI-generated clinic letters in complex paediatric neurosurgery outpatient settings

**DOI:** 10.1007/s00381-026-07171-6

**Published:** 2026-02-19

**Authors:** Mohamed Elmolla, Anjum Aarifa Khanom, Ahmad M. S. Ali, Christian Duncan, Anusha Hennedige, Vejay N. Vakharia

**Affiliations:** 1 The Walton Centre NHS Foundation Trust, Liverpool, UK; 2 Aintree University Hospital, Liverpool, UK; 3 Alder Hey Children’s NHS Foundation Trust, Liverpool, UK; 4 The Department of Cancer Medicine, Institute of Systems, Molecular and Integrative Biology, University of Liverpool, Liverpool, UK

**Keywords:** Generative artificial intelligence, Clinic letters, Natural language processing, Health communication

## Abstract

**Purpose:**

Dictation of outpatient clinic letters can result in increased workload for clinicians. The use of generative, natural language processing artificial intelligence (AI) software could be used to supplant dictation and typing, alleviating the clinician’s workload. Therefore, our objective was to validate the use of AI software, Lyrebird AI (Lyrebird Health, Ltd.) to create accurate and readable clinic letters, in the context of a single clinician general paediatric neurosurgery clinic and a multi-disciplinary craniofacial clinic.

**Methods:**

Twenty consultations were included, wherein a microphone was used to record the entire consultation. For each consultation, two letters were generated independently: (1) Lyrebird AI letter automatically generated at the end of the recording and (2) human clinician manual dictation in the usual manner. The letters were compared using objective readability metrics and a subjective rating by an independent blinded clinician for clinical accuracy.

**Results:**

AI-generated clinic letters significantly improved readability compared to clinician-dictated letters, when using the Flesch–Kincaid Grade Level (median 10.3, IQR 0.75), (median 10.9, IQR 1.15) (*z* = 2.73, *p* < 0.05), and SMOG Index (median 11.7, IQR 1.25) (median 13.1, IQR 0.9) (*z* =  − 3.36, *p* < 0.001) metrics. An independent blinded clinician subjectively chose the AI-generated letters for overall preference in 75% of cases.

**Conclusions:**

Lyrebird AI–generated clinic letters increased readability whilst maintaining clinical accuracy. Future work should focus on time, effort, and cost saving analysis. Presently, the findings of this study provide validation of Lyrebird AI for complex, multi-stakeholder clinical settings.

**Supplementary Information:**

The online version contains supplementary material available at 10.1007/s00381-026-07171-6.

## Introduction

Accurate and comprehensible communication between clinicians and patients is essential. In the outpatient setting, clinic letters are used to summarise discussions and are an important record of the patient’s clinical history, diagnosis, and treatment plan. Presently, dictation, which requires human transcription and checking, is used to create clinic letters. This has been shown to be an inefficient use of a clinician’s time, resulting in increasing stress and job dissatisfaction [1–4]. Additionally, it can also add delay to the letter reaching both the patient and other clinicians involved with the management.

Whilst conventional voice recognition software can be used to mitigate typing, it requires dictation and remains inaccurate due to its inability to process, learn from, and understand input. In comparison, novel generative artificial intelligence (AI) or natural language processing (NLP) tools can be used to automate the generation of clinic letters without the need for any formal dictation simply by passively ‘listening’ to the consultation. This reduces overall consultation times and cognitive burden for the clinician, but it is essential that the text output is clinically accurate without compromising readability and language nuance [5, 6].

With the rapid progression in artificial intelligence (AI) and the development of large language models (LLMs), commercial software is now available that can summarise pertinent information from the consultation without the need for the clinician to dictate a letter. By passively listening in to the consultation, the software can distinguish the healthcare professionals from the patient and family members, extract clinically useful information from the consultation, and summarise the information in a letter format that has been predefined as a template. To determine if such models are practical and accurate enough for use in a complex outpatient setting, we undertook a validation study including both a single clinician general paediatric neurosurgery clinic and a multi-disciplinary craniofacial clinic.

## Methods

A prospective case–control study was conducted over three consecutive clinics, using Lyrebird AI (Lyrebird Health, Ltd.), as it is the NLP tool being trialled at our institution.

In this study, the outpatient clinic runs in the typical manner—the clinician sees the patient who attends with their family member(s). The clinician also opens the Lyrebird application at the beginning of the appointment, which records the consultation via a dictaphone that is placed on the table. Verbal consent was taken at the start of the consultation prior to starting the recording in line with the current trust usage policy for Lyrebird. Following this, Lyrebird assimilates the conversation in real time and generates a letter immediately as the appointment concludes. The Lyrebird letters can be edited post-hoc but were not for the purposes of this study. Separately to this and in a blinded manner, the clinician retrospectively dictates their letter in the usual fashion, before it is sent for typing. Two letter templates with different, appropriate formatting were set up prior for the general neurosurgery and craniofacial clinics. All data was anonymised, and all letters were saved in the hospital’s electronic patient record.

For the general neurosurgery clinic, there was one clinician, one patient aged 0–18 years old, and 1–4 family members. In contrast, the MDT clinic contained eight clinicians (comprising a neurosurgeon, plastic surgeon and/or maxillofacial surgeon, speech and language therapist, geneticist, dentist/orthodontist, specialist nurse, and a clinical psychologist), one patient aged 0–15 years old, and 1–2 family members.

The study included twenty consultations. A comparison between the Lyrebird AI–generated and clinician-dictated letters was then undertaken, using four objective metrics of readability (see supplementary materials for further information):

1. The Flesch–Kincaid Grade Level (FKGL) uses the total number of syllables, words, and sentences to derive the approximate age of schooling needed to understand a given piece of text [7]. A higher score confers a higher level of schooling required.
2. The Flesch Reading Ease (FRE) was used to assess the overall readability of the clinic letters, in which scores are similarly calculated using the total number of syllables, words, and sentences [8]. Scores span a scale of 1–100, with higher scores conferring easier readability.
3. The Gunning Fog Index (GFI) uses total words, sentences, and word complexity to assess the overall complexity of text [9]. On a scale of 0–20, higher scores indicate higher complexity.
4. The Simple Measure of Gobbledygook (SMOG index) was used to assess the simplicity of a text by considering the polysyllabic word count; higher scores relate to a higher level of schooling required to read the text [10].

Human rater Likert scale (1–5) assessment was also undertaken by one expert, independent blinded clinician rater—here, they reviewed letters for content accuracy and comprehensiveness, captured as their overall subjective preference. Statistical analysis, using Wilcoxon Signed Rank test, was completed in R (version 4.4.2); a *p* < 0.05 was deemed statistically significant.

## Results

All 20 consultations were analysed by both statistical analysis and human rater assessment. As all data were not normally distributed, non-parametric Wilcoxon signed rank test and descriptive statistics (median, interquartile range [IQR]) were used.

### Quantitative analysis

When using the FKGL metric, the Lyrebird AI–generated clinic letters (median 10.3, IQR 0.75) demonstrated higher readability than the clinician-dictated letters (median 10.9, IQR 1.15) (*z* = 2.73, *p* < 0.05). Similarly, the SMOG Index demonstrated that the Lyrebird AI–generated letters (median 11.7, IQR 1.25) were more readable than the dictated letters (median 13.1, IQR 0.9) (*z* =  − 3.36, *p* < 0.001) (Table 1). There was a non-significant difference between the types of letters when assessing them via the GFI (*z* =  − 1.22, *p* = 0.22) and the FRE indices (*z* =  − 0.84, *p* = 0.40) (Table 1).

**Table 1:** Summary of metrics

	**Flesch–Kincaid grade level**		**Gunning fog index**		**SMOG index**		**Flesh reading ease**		**Expert clinical rater–Likert scale scores & preference**		
**Subject**	**AI**	**Clinician**	**AI**	**Clinician**	**AI**	**Clinician**	**AI**	**Clinician**	**Lyrebird letter score**	**Clinician letter score**	**Rater’s preference**
1	10.0	10.9	11.8	11.7	11.6	13.1	43.3	41.7	5	3	LB
2	8.4	11.4	11.3	12.9	11.5	13.3	54.9	45.2	5	3	LB
3	10.8	11.2	12.6	12.0	12.7	13.5	36.3	43.8	5	4	LB
4	10.5	12.4	14.5	14.0	13.7	14.6	44.8	39.2	5	3	LB
5	7.7	10.2	8.3	10.5	10.3	12.7	57.5	49.6	5	3	LB
6	9.5	11.2	11.7	13.5	11.6	13.5	47.0	44.9	4	4	Unequivocal
7	11.7	10.7	15.2	12.0	14.3	12.7	40.7	46.3	5	3	LB
8	7.5	7.7	10.0	8.9	10.7	10.9	59.2	64.4	5	5	Unequivocal
9	10.0	9.5	12.5	11.0	12.6	12.9	42.0	52.8	5	5	Unequivocal
10	9.4	10.3	11.1	11.7	11.6	12.4	48.0	53.7	4	5	Clinician
11	9.6	11.1	11.8	12.2	11.7	12.8	45.8	44.7	5	4	LB
12	10.4	11.5	13.9	13.9	12.8	13.6	40.4	49.4	4	5	Clinician
13	11.1	12.2	14.4	15.6	13.1	14.4	35.0	31.2	5	4	LB
14	10.3	8.4	10.9	10.5	11.3	11.2	57.5	60.3	5	3	LB
15	10.3	11.0	13.2	13.1	12.7	13.4	37.5	42.9	5	3	LB
16	10.3	10.1	11.7	12.0	11.7	12.3	43.8	46.2	5	2	LB
17	10.3	11.0	11.0	13.0	11.3	13.2	39.8	44.6	5	3	LB
18	10.3	13.8	13.0	15.8	12.9	15.2	35.4	34.6	5	3	LB
19	10.3	11.5	12.1	13.1	12.0	13.7	47.1	44.6	5	2	LB
20	10.3	11.1	11.1	11.7	11.4	12.3	47.9	45.0	5	3	LB
Median	10.3	10.9	11.8	12.5	11.7	13.1	44.3	46.3			
IQR	0.75	1.15	1.95	1.5	1.25	0.9	7.675	5.875			
Wilcoxon signed rank test	*Z*-value = 2.7253		*Z*-value = − 1.2274		*Z*-value = − 3.3599		*Z*-value = − 0.84				
	***p* < 0.05**		***p* = 0.22**		***p* = 0.00078**		***p* = 0.4**				

*IQR* interquartile range, *LB* Lyrebird

### Qualitative analysis

In the human rater assessment, the Lyrebird AI–generated letters were preferred to clinician-dictated letters in 75% (*n* = 15) of cases, clinician-dictated for two letters, and three letters in which there was an unequivocal difference. Both Lyrebird AI–generated and clinician-dictated letters held the same content. Importantly, Lyrebird AI–generated letters did not detail any information that was deemed unsafe or incorrect.

With regards to content, there were three primary differences between the Lyrebird AI–generated and clinician dictated letters.

Firstly, when a clinical examination of the patient was conducted (11 out of 20 consultations), Lyrebird AI–generated letters consistently offered details of the entire examination including negative findings, whereas clinicians dictated only the salient points.

Secondly, all letters formatted a summary of the management plan at the top of the clinic letter, and outside of the main body of text. However, this plan was comprehensive in all 20 of Lyrebird AI–generated letters, by informing patients and relatives of aspects which required no further action, such as “no further routine imaging required”. Clinicians only dictated aspects of management plans that would result in further action in most cases (14 out of 20 letters).

Finally, all letters also formatted a summary of the relevant diagnoses at the top of the clinic letter and outside of the main body of text. In six cases, clinician-dictated letters were more nuanced than the Lyrebird AI letters, containing adjectives that helped allude to the patient’s clinical course, for example: detailing relevant past surgical history; stating “incidental finding of posterior fossa arachnoid cyst” as opposed to “posterior fossa arachnoid cyst”; and describing “generalised cerebral atrophy” as opposed to “cerebral atrophy”, respectively.

## Discussion

### Present study and literature review

In this prospective case–control study, we examined the readability of 20 Lyrebird AI–generated versus 20 consultant-dictated clinic letters. Lyrebird AI was found to significantly improve the readability of clinic letters across 2 of 4 metrics, whilst maintaining clinical accuracy. There was a non-significant difference for the remaining metrics. In addition to objective measures, an independent rater subjectively described an overall preference for the Lyrebird AI–generated letters by a wide margin.

To our knowledge, this is the first paper to directly compare AI-generated and clinician-dictated clinic letters for real patients, wherein the AI software is purpose built for healthcare documentation. Lyrebird AI significantly improved the readability of clinic letters across 2 of 4 metrics, whilst maintaining accuracy. This indicates that the Lyrebird AI–generated letters are able to convey the same complexity of information at a lower reading age. In addition, given the complex nature of specialty neurosurgery and craniofacial clinic there is a certain amount of medical nomenclature such as genetic diagnoses, physical examination and operative procedures that cannot be simplified further, which may explain why differences were only found in two of the four metrics.

Our findings demonstrate that generative AI could be employed to reduce the administrative burden on clinicians. Perhaps one of the most powerful applications of Lyrebird AI is that it can contend with MDT settings to assimilate multiple specialist conversation lines into a concise summary that remains readable to non-healthcare professionals.

When considering these findings, it is important to refrain from generalising all generative AI software into a single entity [11, 12]. The majority of literature has so far been published on the use of the most widely available free chatbot, ChatGPT from Open AI (Table 2) [1, 5, 6, 13–17]. For example, a prospective cohort study carried out in 2024 found that ChatGPT was able to increase readability and maintain the accuracy of orthopaedic surgery outpatient clinic letters [13]. However, like other studies, the authors found the chatbot lacked the same language nuance as clinician-dictated letters, which resulted in sentences that lacked tact and were unnecessarily superfluous [13, 16, 17]. Whilst the current study demonstrates that clinicians do provide more technical nuance in some aspects, particularly in the summary of diagnoses, Lyrebird AI–generated letters generally held the same meaning and did not omit any clinical information that might change the clinical impression or management plan of the patient. This difference may be because Lyrebird AI is a generative AI software that is purpose-built for healthcare documentation.

**Table 2:** Summary of studies to date comparing the qualities of AI software–generated to clinician-generated outpatient letters

Author	Year	Study type	Sample size (*n*)	Setting	AI software	Comparative metrics	Results
Ellmolla et al. (current study)	2024	Prospective case control	20	Real-world Paediatric craniofacial subspecialist outpatients and MDTs	LyreBird (LyreBird Health, Ltd.)	Fleisch–Kincaid grade level	Significant improvement with AI
						SMOG index	Significant improvement with AI
						Gunning fog index	No significant difference
						Flesch reading ease	No significant difference
						Blinded human rater: subjective preference of letter	No significant difference
Bass et al	April 2025	Prospective comparison study	4	Simulated Paediatric ENT same day post-surgical discharge	ChatGPT 3.5 (Open AI, Ltd.)	Blinded human rater: quality of medical information	Significant improvement with AI
						Blinded human rater: ease of reading	Significant improvement with AI
						Blinded human rater: length of letter	No significant difference
Balloch et al. [19]	June 2024	Simulated interventional study	47	Simulated Adult multi-specialty outpatients	Tortus (Tortus AI, Ltd.) ChatGPT 4.0 (Open AI, Ltd.)	Sheffield assessment instrument for letters	Significant improvement with AI
						Total timing of consultation	Significantly shorter consultation with AI
						Total time speaking to patients	No significant difference
						NASA task load index	Significant improvement with AI
						Blinded human rater assessment: overall experience	Significant improvement with AI
Stoneham et al. [13]	April 2024	Prospective cohort study	70	Real-world Adult orthopaedic subspecialist outpatients	ChatGPT 3.5 (Open AI, Ltd.)	Mean word count	Significant increase with AI
						Flesch reading ease	Significant worsening with AI
						Flesch–Kincaid grade level	Significant worsening with AI
					ChatGPT 4.0 (Open AI, Ltd.)	Mean word count	Significant increase with AI
						Flesch reading ease	No significant difference
						Flesch–Kincaid grade level	No significant difference

### Future work

The potential of Lyrebird AI could be realised further by exploring its text output in a context-dependent manner [6]. It has potential for use in settings in which patients are not participants—for example, to summarise decisions made in inter-specialty multi-disciplinary meetings. Additionally, Lyrebird AI has the function to create several custom formatting templates that the clinician can preset. Individual specialties, and indeed individual clinicians, will have preference for clinic letter formatting in order to highlight essential information. The impact of template refinement should be investigated as a further potential time-saving avenue, and for enhancing user experience through individualising the software for each healthcare setting.

These considerations ultimately implicate future work on economic analysis of generative AI use in the clinical setting. The market for AI tools with healthcare applications is large and growing exponentially larger [18]. By nature of such a market, each individual product will require its own cost-efficacy validation, which remains a barrier, but is essential for wide-scale uptake. It remains imperative that such tools are rigorously validated, and clinical letters are checked for factual content and accuracy by clinicians, which is no different from human-generated letters.

### Limitations

The limitations of this work include: (1) the objective metrics used are generalised measures of readability, as healthcare-specific readability metrics do not currently exist to our knowledge; (2) the findings are only applicable to one type of commercially available commercially available generative AI software and are therefore not generalisable; and (3) the clinical context is a highly specialised neurosurgical and craniofacial clinic, which again means the findings may not be generalisable to other settings, such as general practice or the emergency department. These limitations highlight the potential focus of future work. Namely, if generative AI is being used to enhance clinician effort, then analysis of overall time and effort saving is essential to further quantify its efficiency as a tool. Balloch et al. [19] have successfully demonstrated this in simulation, with a view to expand to real-world clinical environments.

## Conclusions

Through the use of both objective readability metrics and subjective blinded clinician ratings, we provide validation of the Lyrebird AI generative software for use in a highly specialist single-clinician general neurosurgery clinic and multi-disciplinary craniofacial clinic environment. Unlike previously described technologies, it does not require the burden of a carefully considered prompt, nor does it require redaction of sensitive patient information. Rather, it is a single software that ambiently listens to clinicians, patients, and families to immediately scribe a readable and accurate letter which can be reviewed and sent without delay. Future work should focus on time, effort, and cost-saving analysis, in addition to template refinement and applications in other clinical settings.

## Supplementary Information

Below is the link to the electronic supplementary material.

Supplementary file1 (DOCX 19 kb)

## Data Availability

No datasets were generated or analysed during the current study.
