# Supplementary material for: Integration of AI-generated clinic letters in complex paediatric neurosurgery outpatient settings
Source: Childs Nerv Syst. 2026 Feb 19;42(1):80. doi: 10.1007/s00381-026-07171-6 (PMC12917027; doi:10.1007/s00381-026-07171-6)
Supplement: Supplementary file 1 — Supplementary file1 (DOCX 19 kb) [file 381_2026_7171_MOESM1_ESM.docx]

**Lyrebird-AI Supplementary Material**

**Readability Metrics**

FKGL —> 0.39 ( Total Words / Total Sentences ) + 11.8 (Total Syllables / Total Words) − 15.59

FRE —> 206.835 − 1.015 (Total Words / Total Sentences) − 84.6 (Total Syllables / Total Words)

GFI —> 0.4 ((total words / total sentences) + 100(total complex words / total words))

SMOG —> 1.043 √((polysyllabic words) (30/sentences) + 3.1291)

**Lyrebird-AI Letter Template**

1. Diagnosis

List the main diagnoses or suspected conditions. Use numbered points if there are multiple diagnoses. Be concise but specific, including any relevant timeframes or dates. Include the genetic diagnosis including stating if no genetic diagnosis has been found

Sentence Structure: Numbered

Length of section: Regular

1. Operations

Include any operations the patient has had with the dates and any operative findings

Sentence Structure: Numbered

Length of section: Regular

1. History

Begin with 'It was a pleasure to review [child's name] with [their parent/guardian] in the General Paediatric Clinic.' Provide a detailed account of the patient's history, including presenting complaints, duration of symptoms, and any relevant past medical history. Use paragraphs to separate different aspects of the history.

Sentence Structure: Paragraph

Length of section: Longer

1. Investigations:

List all relevant investigations, including dates and results. Use numbered points. Include both completed and pending investigations. Be specific about timeframes for future tests.

Sentence Structure: Numbered

Length of section: Regular

1. Examination:

Describe the physical examination findings in detail. Include head circumference with centiles as well as anterior-posterior distance, biparietal distance, bifrontozygomatic distance, left oblique and anterior oblique distances. Describe the overall head shape.

Sentence Structure: Dot-points

Length of section: Regular

1. Planned procedures

Include if the patient is due to undergo surgery as well as the operative details, risk and benefits of surgery as well as alternative operative options as part of informed consent. Provide as much detail as possible including percentage estimates of risk and future potential complications. State if surgery has been offered as well as what other options are including conservative management.

Sentence Structure: Dot-points

Length of section: Longer

1. Follow-up:

Briefly state the follow-up plan. Include whether the patient is being discharged or when they will next be seen.

Sentence Structure: Paragraph

Length of section: Regular

1. Summary and Plan:

Begin with 'In Summary,'.

Summarise the key points of the consultation, including main findings and diagnoses. Outline the management plan, including any follow-up arrangements or further investigations.

Sentence Structure: Paragraph

Length of section: Regular

1. Yours sincerely

End the letter with 'Yours sincerely' followed by [Clinician Name and Designation]'.
